# Legal and Ethical Considerations for the Design and Use of Web Portals for Researchers, Clinicians, and Patients: Scoping Literature Review

**DOI:** 10.2196/26450

**Published:** 2021-11-11

**Authors:** Michael Lang, Sébastien Lemieux, Josée Hébert, Guy Sauvageau, Ma'n H Zawati

**Affiliations:** 1 Centre of Genomics and Policy, Department of Human Genetics Faculty of Medicine and Health Sciences McGill University Montreal, QC Canada; 2 Department of Medicine Université de Montréal Montreal, QC Canada; 3 The Leucegene Project at Institute for Research in Immunology and Cancer Université de Montréal Montreal, QC Canada; 4 Division of Hematology Maisonneuve-Rosemont Hospital Montreal, QC Canada

**Keywords:** medical ethics, web portal, scoping review, eHealth, portal

## Abstract

**Background:**

This study aims to identify a novel potential use for web portals in health care and health research: their adoption for the purposes of rapidly sharing health research findings with clinicians, scientists, and patients. In the era of precision medicine and learning health systems, the translation of research findings into targeted therapies depends on the availability of big data and emerging research results. Web portals may work to promote the availability of novel research, working in tandem with traditional scientific publications and conference proceedings.

**Objective:**

This study aims to assess the potential use of web portals, which facilitate the sharing of health research findings among researchers, clinicians, patients, and the public. It also summarizes the potential legal, ethical, and policy implications associated with such tools for public use and in the management of patient care for complex diseases.

**Methods:**

This study broadly adopts the methods for scoping literature reviews outlined by Arskey and O’Malley in 2005. Raised by the integration of web portals into patient care for complex diseases, we systematically searched 3 databases, PubMed, Scopus, and WestLaw Next, for sources describing web portals for sharing health research findings among clinicians, researchers, and patients and their associated legal, ethical, and policy challenges. Of the 719 candidate source citations, 22 were retained for the review.

**Results:**

We found varied and inconsistent treatment of web portals for sharing health research findings among clinicians, researchers, and patients. Although the literature supports the view that portals of this kind are potentially highly promising, they remain novel and are not yet widely adopted. We also found a wide range of discussions on the legal, ethical, and policy issues related to the use of web portals to share research data.

**Conclusions:**

We identified 5 important legal and ethical challenges: privacy and confidentiality, patient health literacy, equity, training, and decision-making. We contend that each of these has meaningful implications for the increased integration of web portals into clinical care.

## Introduction

### Background

Medical care and health research are jointly undergoing significant changes brought about by the internet [1-3]. New web portals, apps, and programs are helping to facilitate unprecedented levels of data sharing and collaboration, potentially enabling more precise targeted treatment and rapid research translation [4-6]. Web portals have been a significant part of this emerging web-based health ecosystem, providing patients with a mechanism for accessing electronic health records, managing appointments and prescriptions, and communicating directly with care providers [7]. Much has been written about the technical and ethical challenges associated with the development and integration of web portals into clinical settings [8,9]. However, portal technology might also be used to connect health researchers to clinicians, patients, and the public. Web portals, for example, could be a useful platform for broad and rapid dissemination of research results. Although the most prevalent and widely discussed types of web portals are those used to manage patient interactions with the health care system (so-called *patient portals*), other potential uses are being increasingly identified.

### Objective

This study aims to identify one such novel potential use: the adoption of web portals to rapidly share health research findings with clinicians, scientists, and patients. In the era of precision medicine and learning health systems, the translation of research findings into targeted therapies depends on the availability of big data and emerging research results. Web portals may work to promote the availability of novel research, working in tandem with traditional scientific publications and conference proceedings. Web portals raise the possibility that important research findings will be shared efficiently not only with scientists and clinicians but also with patients and members of the public. This study aims to examine how web portals can be used to facilitate such sharing. We seek to preliminarily outline how web portals can be used to advance research sharing objectives and to identify legal and ethical barriers that might challenge these functions. We will distinguish between *web portals* as a general category, by which we mean web resources that aggregate health information for a specific purpose, and *therapeutic portals*, by which we mean web portals designed to aggregate health research findings and make them available to a diverse array of researchers, clinicians, and the general public. Therapeutic portals may be contrasted with one prominent type of web portal, the *patient portal*. This is a system typically used to share individual patient data, facilitate clinical interaction, and record specific health outcomes [10]. For the purposes of this study, we propose the concept of a *therapeutic portal* in an attempt to capture nuances that are not fully expressed in discussions on the use of web portals for managing and sharing health research findings. In the era of precision medicine and learning health systems, as interactions between clinicians, researchers, and the public take on a unique and pressing character, we are of the view that the language of therapeutic portals better fits the potential emerging use of portal technology in these contexts. Although the distinction between these kinds of web portals is subtle, it has potentially important implications for the provision of care and for the legal and ethical obligations of clinicians in health care systems increasingly modulated by the internet.

Our interest in studying the feasibility of therapeutic portals is motivated by an ongoing multidisciplinary molecular genetics research project to better characterize and categorize the incidences of acute myeloid leukemia (AML) [11]. The Leucegene project is a biobank-based study adopting next-generation sequencing, chemotherapeutics, and precision-medicine approaches for identifying prognostic markers and therapeutic targets for AML [12]. Research in this area, which brings together the efforts of researchers, clinicians who apply novel prognostic testing regimes, and patients who ultimately benefit from AML treatments derived from novel precision-medicine therapies, is fertile ground for exploring the use of internet-mediated tools for widely sharing advancements in the treatment and study of AML. Although using such systems promises to maximize the potential exploitation of new knowledge by making it accessible to as wide an audience as possible, it also raises difficult practical and ethical difficulties.

In this regard, this review aims to explore the potential use of web portals that work to share research results in the context of complex diseases with the research community, clinicians, and patients. In particular, we set out to understand whether such tools are presently in use, how they may be implemented, and how we might account for the pressing policy challenges they raise. This study will help contextualize and support the development of web portals intended to promote information sharing in health care systems by increasingly applying precision-medicine approaches to combat complex diseases. This review examines the potentially expanding scope and utility of web portals and highlights the policy challenges associated with this expansion. This will be achieved by outlining the results of a scoping literature review attempting to ascertain the extent to which web portals have been considered to facilitate communication between researchers and clinicians. Therapeutic portals are internet-enabled tools for sharing information between health researchers conducting basic science research and clinicians implementing best health practices. Tools of this kind expand on the increasing reach of patient portals by broadening their scope of application and inflating their role in patient care. Although there is a sense in which the use of web portals for sharing health research and disease data among clinicians and investigators is a logical extension of the form and function of existing tools that bring together patients and care providers, little direct consideration of such platforms appears to be present in the literature. This review poses 2 questions: (1) Are therapeutic portals being developed and used in health systems? (2) What legal and ethical issues might be raised by the use of therapeutic portals for sharing health research and disease data with researchers, clinicians, and the general public? This study will address these questions in 3 sections. First, we outline the methods used to preliminarily survey the literature in consideration of the questions above. Second, we summarize our results and describe how they apply to the development and use of therapeutic portals. Third, we discuss our findings with a broader literature on web portals and health research dissemination. We also note the limitations of our research and suggest that more work on this topic is needed.

## Methods

We designed this scoping literature review following the theoretical model set out by Arskey and O’Malley [13]. Scoping reviews are a social research methodology that aim to rapidly map *the key concepts underpinning a research area and the main sources and types of evidence available* [14]. Scoping reviews are typically contrasted with more extensive systematic reviews. This study, following Arskey and O’Malley, is intended to identify the broad contours of existing literature and note the gaps requiring further attention [13]. We adopted an iterative search process comprising 5 stages. First, we developed the following 2 research questions: (1) Are therapeutic portals being developed and used in health systems? (2) What legal and ethical issues might be raised by the use of therapeutic portals for sharing health research and disease data with researchers, clinicians, and the general public? Second, we identified relevant studies by searching web databases. Specifically, we consulted Scopus, PubMed/MEDLINE, and WestLaw Next using fixed Boolean keywords including *online portal* AND *health* AND *communication* AND research* AND clinic*. Our search strategy initially focused on web portals as a general category, noting that scholarly sources describing portals used primarily for communicating research findings to a wide audience were not organized using discretely identifiable terminologies. We refined search terms iteratively and scanned source references to broaden the scope of the review. A primary search was conducted in April 2019, and a final search was conducted in March 2020. We did not time limit our search to obtain the widest possible array of sources. Initially, we identified 719 articles and papers from each source. Third, we selected studies for inclusion according to the criteria developed *post hoc*, as we became increasingly familiar with the literature. Owing to the nature of the research questions, as well as inconsistent language in the literature for explaining the function of web portals, determining which sources to include in our analysis sample proved to require a delicate balance. We worked with a set of flexible inclusion criteria. We included sources that met any one of the following broad criteria: (1) description of a web portal in actual use for the purpose of sharing research findings or non–patient-specific data; (2) description of a potential use case for web portals that share research findings or non–patient-specific data among researchers, clinicians, and the public; (3) description of a potential use case for web portals that permit access to health research findings or lay explanations of health research findings for patients or the general public; and (4) descriptions of legal and ethical issues associated with any of the above criteria. We excluded (1) duplicate sources, (2) sources not available in English, and (3) sources that primarily discuss the use of web portals for purposes other than the sharing of health research. Exclusion criterion 3 resulted in a large number of sources being screened out. In particular, numerous sources focused narrowly on the development and implementation of *patient* portals were generally not included in the review. Of the 719 sources initially collected, 22 (3%) sources were retained for the final review. Fourth, we charted source data for each retained resource, including the author, year of publication, and conclusions. These data are outlined in Table 1. Fifth, we summarized and reported the results of the review in this study.

## Results

### Initial Screening

**Figure 1:**
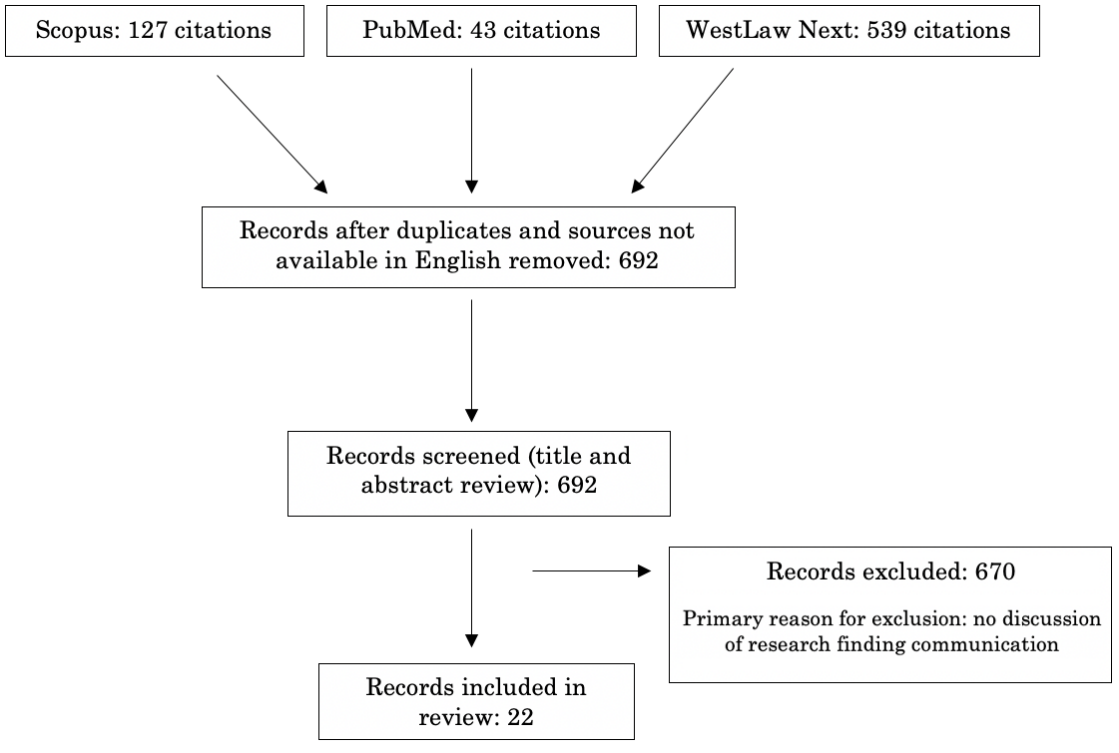
Scoping review flowchart.

We selected 22 sources for the final review. Of the 719 sources initially obtained, 25 (3%) were eliminated as duplicates and 2 (0.2%) were unavailable in English. This left 96.2% (692/719) of papers for which we conducted a title and abstract review. Of these, we found that a high number primarily outlined work on the adoption of patient portals. Following exclusion criteria 3 above, those sources that did not include discussion about the potential communication of research findings, in addition to patient data such as testing results and treatment plans, were generally determined to fall outside the ambit of this review. We eliminated 93% (671/719) of sources that were determined not to meet the inclusion criteria described above. Operating on a relatively narrow conception of therapeutic portals, we did not expect to find a large number of sources that would meet the inclusion criteria outlined above. Nevertheless, we determined that the 3% (22/719) included sources provide an overview of this emerging field and suggest that the adoption of web portals for sharing research findings will become increasingly prominent in the coming years. This limited overview of the early literature in this space may work to ground subsequent discussions. Figure 1 outlines the steps taken to conduct this review.

### Study Selection

The sources retained for the final review were relatively varied in their theoretical and methodological orientations. Several studies, for example, described patient portals enhanced by the inclusion of health data that would not be included in patient charts. One such approach is exhibited in a work by Nordqvist et al [15], who outlined the attitudes of health professionals on a patient portal supplemented with disease-specific information intended to promote adolescent diabetes care. Other articles documented the creation of a novel web portal for safely sharing participant-level data with researchers [16,17] or an open web portal designed to facilitate the sharing of gene association data for lung cancer [18]. Other records included in our final sample mentioned issues surrounding research data sharing somewhat less directly [19]. Following the abstract and title review, we determined that these sources warranted inclusion in the final analysis. Where the precise contours of a proposed or existing portal were not clearly delineated, we erred on the side of the inclusion. This led to the analysis of a number of articles that do not directly contemplate what we have described as therapeutic portals, but that *do* contemplate use cases to facilitate access to health research findings. We believe that these sources fall under inclusion criteria 2, describing a potential use case for web portals that share research findings or non–patient-specific data among researchers, clinicians, and the public. We explore these themes in greater detail in the *Discussion* section.

### Results Charting

We charted our overarching results in Table 1, which summarizes findings derived from sources reviewed at this final stage and conveys our determination on whether these sources describe tools that could reasonably be considered therapeutic portals. Of the 22 references, we established that 8 (36%) contemplated a web resource to facilitate some form of communication among researchers, patients or the public, and clinicians. The manner in which these resources describe therapeutic portals varies considerably.

**Table 1:** Methodological and theoretical orientations of reviewed articles.

Study and article title	Article objective	Article conclusions	Therapeutic portal
Baldwin et al [19], *Patient portals and health apps: Pitfalls, promises, and what one might learn from the other symptoms*	Limitations and potential of 2 kinds of patient-facing information technology: portals and applications	Combining features of mHealth apps and portals could increase patient engagement	No
Bartonova [20], *How can scientists bring research to use: The HENVINET experience*	Describes HENVINET, a portal for sharing research findings among scientists, policy makers, and the public	Portal highlights the need for liaison between researchers, policy makers, and the public	Yes
Bostrom et al [21], *Strategic and integrated planning for healthy, connected cities: Chattanooga case study*	Describes a web portal combining location and health data to identify areas for potential greenspace development	Portal determined access to park space. Promotes integrating community and social metrics to equitably address public health challenges	No
Bowler et al [22], *The visibility of health web portals for teens: A hyperlink analysis*	Assesses teen health websites for accessible and reliable health information	Websites had a low level of visibility relative to resources intended for other audiences. Information for teens present on resources that lack health expertise	Yes
Ling Cai et al [18], *LCE: An open web portal to explore gene expression and clinical associations in lung cancer*	Discusses a lung cancer database with expression and clinical data from 6700 patients in 56 studies	The Lung Cancer Explorer is publicly accessible and provides genomic and tissue image data for lung cancer	Yes
Christensen et al [23], *Beacon: A web portal to high-quality mental health websites for use by health professionals and the public*	Describes the Beacon web portal, which aggregates lists of high-quality health websites sharing information on mental health	There are many high-quality web resources available for mental health. The Beacon portal attempts to identify and gather them in a single resource	Yes
Das et al [24], *The impact of an eHealth portal on health care professionals’ interaction with patients: qualitative study*	Discusses implementation of a web portal intended to help weight loss surgery patients achieve healthy outcomes	Implemented eHealth portal was a valuable source of information and a gateway for facilitating positive patient interactions	No
Kaiser [16], *A new portal for patient data*	Presents the Vivli portal, which is intended to support the sharing of anonymized clinical study data	The portal makes available the results of more than 4000 clinical trial data sets from 8 companies and nonprofits	No
Kirkpatrick et al [25], *GenomeConnect: matchmaking between patients, clinical laboratories and researchers to improve genomic knowledge*	Presents the GenomeConnect portal, which provides a space for patients and members of the public to share health history and genetic test results	GenomeConnect portal allows members of the public to participate in genetics research and to contribute to the validation of novel clinical tests	Yes
Kohler [26], *Can internet access growth help reduce the global burden of noncommunicable diseases?*	Describes an open access portal for linking disparate source health information for reducing preventable lifestyle-related risk factors associated with noncommunicable disease	Web portals of the kind described have the potential to improve the global burden of noncommunicable disease if implemented at scale	No
Kuijpers et al [27], *An interactive portal to empower cancer survivors: a qualitative study on user expectations*	Studies the perspectives of cancer survivors on the possible features of an interactive web portal	Participants interested in portal features that fulfill information needs, such as access to their eHealth record	No
Li et al [17], *Moving data sharing forward: the launch of the Vivli platform*	Presents the Vivli portal for supporting anonymized clinical study data sharing	Data sharing portals have an important role to play in addressing issues around reidentification and anonymization	No
Maggio et al [28], *Qualitative study of physicians’ varied uses of biomedical research in the USA*	Perspectives of physicians on interaction with biomedical research presented on a web portal	Physicians reported a high level of research adoption and appealed to their multi-faceted roles as clinicians, educators, and researchers	No
Marrie et al [29], *Use of eHealth and mHealth technology by persons with multiple sclerosis*	Describes use of eHealth and mHealth systems by patients with multiple sclerosis	Internet-enabled tools help to facilitate the sharing of health information between clinicians and patients with multiple sclerosis	No
McKemmish et al [30], *Consumer empowerment through metadata-based information quality reporting: the breast cancer knowledge web portal*	Describes development of the BCK–Web portal, a web resource for sharing health information with patients with breast cancer	BCK–Web portal communicates high-quality medical and experiential knowledge	Yes
Melchart et al [31], *Introduction of a web portal for an individual health management and observational health data sciences*	Establishes a set of core objectives and processes implementing a web portal for lifestyle changes and individual health management	Web tools help to facilitate individual health management in concert with health coaching	No
Melholt et al [32], *Cardiac patients’ experiences with a telerehabilitation web portal: implications for eHealth literacy*	Explores use of a web portal cardiac patient rehabilitation. Outlines health literacy effects	A web portal for rehabilitation among cardiac patients may increase health literacy	No
Nordfeldt et al [33], *To use or not to use – practitioners’ perceptions of an open web portal for young patients with diabetes*	Documents clinician perspectives on the use of an open access web portal for patients with juvenile diabetes	Clinicians felt comfortable recommending web resources for which available information was verifiably reliable	Yes
Nordqvist et al [15], *Health professionals’ attitudes towards using a web 2.0 Portal for child and adolescent diabetes care: qualitative study*	Describes clinician perspectives on the use of a *Web 2.0* portal for juvenile patients with diabetes	Clinicians exhibited positive attitudes toward the portal. Support close collaboration between stakeholders in the development of future portals	Yes
Rocker [34], *Use of a web portal to facilitate clinical trial recruitment: preliminary analysis of fox trial finder*	Characterizes research volunteers registered on a web portal for clinical trial participation recruitment for the study of Parkinson’s disease	Persons affected by Parkinson’s disease willing to participate in health research and share personal data on the web	No
Sutherland et al [35], *A novel open access web portal for integrating mechanistic and toxicogenomic study results*	Describes collaborative toxicogeomics, a web portal for sharing *best practice* methods in computational biology	The developed open-source portal helps to increase accessibility, transparency, and collaboration between researchers in the field	No
Tomlinson et al [36], *MiMiR – an integrated platform for microarray data sharing, mining and analysis*	Presents MiMiR, a web portal supporting the management and sharing of microarray data	MiMiR portal contains more than 150 data points and over 3000 hybridizations supporting the microarray user community	No

Table 2 outlines the general thematic orientations of each of the 8 papers that we determined to have directly contemplated therapeutic portals. Beyond thematic diversity, we also found that reviewed sources considered a wide array of potential policy issues raised by the adoption of web portals in health care, both in the case of therapeutic portals and that of similarly disposed web portals. In particular, we found that 5 legal, ethical, and social issues were raised in the 22 resources we reviewed: (1) privacy and confidentiality, (2) patient health literacy, (3) equity, (4) training, and (5) decision-making. Overall, we determined that out of the 22 resources, 9 (41%) discussed privacy and confidentiality issues, 9 (41%) discussed patients’ health literacy, 4 (18%) discussed equity, 9 (41%) discussed training, and 10 (45%) discussed decision-making. Table 3 outlines the distribution of these dominant policy issues among the reviewed sources.

**Table 2:** Thematic orientation of reviewed therapeutic portals (n=8).

Theme	Resources, n (%)	Study
Genomics	2 (25)	Cai et al [18]; Kirkpatrick et al [25]
Cancer	2 (25)	Cai et al [18]; McKemmish et al [30]
Diabetes	2 (25)	Nordfeldt et al [33]; Nordqvist et al [15]
Mental health	1 (12.5)	Christensen et al [23]
Teenage health	1 (12.5)	Bowler et al [22]
Health and the environment	1 (12.5)	Bartonova [20]

**Table 3:** Distribution of legal, ethical, and social issues (n=22).

Theme	Resources, n (%)	Citations
Privacy and confidentiality	9 (41)	Baldwin et al [19]; Bowler et al [22]; Das et al [24]; Kaiser [16]; Kirkpatrick et al [25]; Kuijpers et al [27]; Li et al [17]; McKemmish et al [30]; Melchart et al [31]
Patient health literacy	9 (41)	Baldwin et al [19]; Das et al [24]; Kirkpatrick et al [25]; Kohler [26]; Kuijpers et al [27]; McKemmish et al [30]; Melchart et al [31]; Melholt et al [31]; Nordqvist et al [15]
Equity	4 (18)	Bostrom et al [21]; Marrie et al [29]; McKemmish et al [30]; Melchart et al [31]
Training	9 (41)	Baldwin et al [19]; Bartonova [20]; Christensen et al [23]; Das et al [24]; Kuijpers et al [27]; Maggio et al [28]; Melchart et al [31]; Nordfeldt et al [33]; Nordqvist et al [15]
Decision-making	10 (45)	Bartonova [20]; Bowler et al [22]; Cai et al [18]; Christensen et al [23]; Das et al [24]; Kirkpatrick et al [25]; Kohler [26]; Kuijpers et al [27]; Maggio et al [28]; Melchart et al [31]

## Discussion

### Principal Findings

#### Overview

This review demonstrates that web portals are presently being used for a wide range of functions and in a variety of clinical and research settings. Ling Cai et al [18], for example, described the Lung Cancer Explorer, a database developed by researchers at the Southwestern Medical Center, University of Texas. The resource houses genomic expression and clinical data on lung cancer and is accessible to the public. Although information included on the Lung Cancer Explorer portal is a highly sophisticated technical data, it is openly accessible and can be used by a public audience attempting to learn about the genomic dimensions of lung cancer. As the third-party processing of raw consumer genetic data becomes more common [37], there may be an increase in the use of portals in the form of Lung Cancer Explorer by members of the public. Research conducted by Leanne Bowler et al [22] investigated an entirely different kind of portal, though one that combines clinical information, health research, and engages members of the public. This work details 6 web portals that provide health information to teenagers, finding that much of the information offered is of dubious quality. Two of the studies described portals for sharing genomic data, two described portals for cancer, and two for diabetes. The remaining themes included mental health, teenage health, and the intersection of health and the environment. We found that therapeutic portalsweb tools that facilitate the sharing of research findings with clinicians, researchers, and patientsare an emerging trend in health care, although they have not yet received widespread attention in the literature.

Notably, we have also found that therapeutic portals have generally not received attention as a phenomenon that is conceptually distinct from the emergence of the patient portal. An extensive literature has been published on the development and use of patient portals in recent years [38], as well as on their capacity to promote patient empowerment and self-care [39]. A growing consensus suggests that the use of a patient portal tends to positively influence health outcomes for patients with chronic disease, despite ongoing concerns about the willingness of such patients to engage with web portals [40]. Patient portals are generally thought to reflect a positive development in health care [41], even as they are accompanied by a range of pressing challenges. The popularization of patient portals has been associated, for example, with an increasing concern about the protection of sensitive or confidential patient data [42]. Our review indicates that web portals are being used for functions that generally fall outside of the scope of patient portals, as they are traditionally conceived. In particular, the increased sharing of basic research results and the expansion of portal audiences to include the general public may raise unique and unforeseen challenges. We believe that these changes may warrant a conceptual distinction between patient and therapeutic portals. As the latter becomes more prominent, it is important that they be subjected to increased scrutiny on their own terms.

Our findings suggest that therapeutic portals are likely to raise a number of important legal, ethical, and social issues. In particular, our review highlights 5 particularly prominent issues: (1) privacy and confidentiality, (2) patient health literacy, (3) equity, (4) training, and (5) decision-making. In this section, we outline how each of these issues is contemplated in our review and suggest policy and regulatory approaches that may be considered to address them.

#### Privacy and Confidentiality

The treatment of privacy and confidentiality issues in the sources identified in this review focused largely on the necessity of ensuring that personal data stored on a web portal is protected, and that data made available to third parties through a web resource cannot be used to identify individual research participants or patients [25]. One source discussed the importance of accounting for privacy regulations in the design of web portals that connect to personal health records [19]. Although therapeutic portals are unlikely to connect directly to personal health records, it is conceivable that they may display individualized or improperly anonymized data. A particular challenge may be determining whether a therapeutic portal is subject to local privacy regulations or is primarily governed by an internal privacy policy. Portals developed in jurisdictions with less privacy protection and made available in others may raise additional privacy considerations. Das et al [24] pointed out that certain web portals may require secure log-in procedures, including specific credentials and passwords, to protect patient information. Not surprisingly, some sources communicated empirical findings that a number of web portal users are concerned about data security and privacy [27]. Issues of privacy and confidentiality are likely to be important considerations wherever sensitive health data are processed or shared [17], including the case of therapeutic portals. With this in view, Melchart et al [31] stress the important role project governance and strong privacy protection principles can play in advancing health research. The overall treatment of privacy and confidentiality issues in the reviewed sources helps to underscore the consensus in the literature that personal information must be carefully safeguarded, especially when such information interacts with internet-enabled tools.

#### Patient Health Literacy

Several articles assessed in this review described the potential health literacy and patient health literacy functions of publicly available web portals for managing and interacting with health data. Indeed, 2 of the 9 papers we found to have discussed patient health literacy, those by McKemmish et al [30] and Kuijpers et al [27], engaged explicitly with empowerment as the orienting theme of their respective contributions. McKemmish et al [30] described a web portal for sharing resources on breast cancer, writing that the *ability to access timely, relevant, and reliable information is a vital component in patient empowerment*. Access to high-quality health information can improve patient outcomes by *facilitating decision-making* and *better treatment compliance* [30]. Similarly, Kuijpers et al [27] outlined the high informational needs of cancer patients and survivors, writing that “it seems imperative to provide cancer survivors with the knowledge, skills, and motivation to positively influence their health status, which is commonly referred to as patient empowerment.” Importantly, Kuijpers et al [27] noted that informational web programs can maximize their empowering potential if accompanied with face-to-face support, suggesting that web portals should not be expected to promote empowerment on their own. Other authors have identified the empowering potential of web portals in passing [15], whereas others focus on the related concept of health literacy. For example, Melholt et al [32] focused on the capacity of web portals to increase health literacy [32]. On the terms of this paper, *eHealth literacy* is conceived as “the ability to seek, find, understand, and appraise health information from electronic sources and apply the knowledge gained to address or solve a health problem” [32]. In this way, the relationship between health literacy and patient empowerment can easily be discerned. Tools that improve a user’s understanding of their health, for example, can be expected to improve outcomes [31,32]. Sources contemplating health literacy and empowerment in this review coalesce with the view that web portals, properly implemented, may positively contribute to both of these outcomes. Critically, however, the authors note that web portals must exist within a supportive health infrastructure, offering reliable and accessible information to achieve these empowering objectives.

#### Equity

One possible effect of increased health literacy facilitated by web resources is the generation of greater equity in access to care. Several studies alluded to this point, although we did not find extensive discussion of health equity promotion in the sample of resources we reviewed. Melchart et al [31], for example, mentioned the importance of designing web systems that are attentive to equality of access concerns, noting that users of a service should be able to depend on receiving high-quality and equitable treatment. Similarly, Bostrom et al [21] described the development of a portal for enhancing greenspace and improving public health specifically to ensure more equitable access to recreational opportunities for underserved populations. Although this study describes a web portal significantly different from those with which we are primarily interested here, it reveals the important role that health information, collected and processed with the help of web systems, can play in advancing health equality. However, the promotion of health equity using web resources is not straightforward. Marrie et al [29] pointed out that web resource use is likely to vary substantially across sociodemographic cohorts. Technology adopters tend to be younger, more highly educated, wealthier, and have higher comorbidities. This generates a serious problem if resource developers intend for web portals to contribute to the advancements in health equity. Ensuring that such portals are accessible to everyone is almost certain to be a perennial challenge, one that is, however, necessary for achieving overarching objectives in this space, including patient empowerment and broader access to leading health research.

#### Training

Further challenging the implementation of novel web health resources is the issue of training. Several of the sources we reviewed detailed the particular training challenges associated with the development of tools intended to be used by clinicians, researchers, patients, and the public. For example, Das et al [24] emphasized the need to ensure that health system personnel feel adequately prepared to effectively use any new web system. Maggio et al [28] made a related point, arguing that little is known about how clinicians access and apply research findings in their practice, which has important *implications for educators designing physician training and policymakers considering public access mandates for research*. Moreover, empirical work suggests that training may be a significant barrier for clinicians to more broadly pursue the use of novel web resources in their practice. Nordfeldt et al [33] wrote that “lack of access, lack of time, and lack of opportunities for training [are] examples of causal factors preventing practitioners from adopting new technologies.” Similarly, researchers accessing web systems may also require contextualizing resources that contribute to the effective use of novel tools. Bartonova et al [20], for example, stressed the importance of training regimes aimed at facilitating the work of research scientists, especially when they are encountering tools that promote interdisciplinary collaboration. In addition, web resources that bring together multiple kinds of stakeholders might also require approaches to training that include patients or the public. Kuijpers et al [27] noted that the use of web health management resources by patients or the public ought to be accompanied by sufficient training and guidance to ensure that such systems are used safely and as intended by their developers. According to Christensen et al [23], the development of web portals for certain serious medical conditions, such as in the mental health context, access to web resources might sometimes need to be accompanied by the ability to consult with trained medical professionals who are able to offer follow-up support and care [23]. Our findings in this review on training considerations are relatively straightforward. Nevertheless, they may have important policy implications. The literature exhibits a strong consensus on the view that training systems should be commensurate with the establishment of novel web portals for health. Such training will ensure that clinicians, patients, and researchers are able to use web portals as effectively as possible, thereby maximizing potential health benefits. As portals continue to be pursued as a means to communicate research findings and general health information among stakeholders, it is critical to develop adequate strategies for training intended users.

#### Decision-making

Our review found that of the 22 sources, 10 (45%) discussed issues related to decision-making. Although this is somewhat an imprecise consideration, in need of greater specificity, it has potentially far-reaching legal, ethical, and policy implications for the use of web portals for health. We discovered that a number of studies gave varying degrees of attention to the ways novel web systems affect clinical and other health-related decision-making. Web resources may affect patient and public decision-making about health simply by influencing the information to which such persons have ready access. Kohler [26] made this point, stating that “information accessed through the internet enables people to make informed decisions about their life and health.” Such resources may also influence medical professionals’ decisions during the course of treatment [27]. The fact that web resources may affect the way health decisions are made is not, by itself, revelatory. It is, indeed, the intended function of such systems to modulate decision-making, hopefully for the more effective and efficient allocation of care resources and for improvements in individual health. However, the precise manner in which such decision-making effects are realized may be critically important. Maggio et al [28] discussed the possibility, for example, that web portals may be used to enable *shared decision-making*, in which therapeutic decisions are reached through dialog between the clinicians and their (informed) patients. Christensen et al [23] noted that certain kinds of persons may be more likely to make medical decisions based on information found on the web than others. They wrote that “consumers living with a disability or chronic disease are more frequent internet users, and more likely to state that they made health decisions based on information found on the web.” Bowler et al [22] similarly found that decisions made by teenage health portal users are often *based on socially constructed content rather content provided by experts in the field*. Although portals have important implications for the manner in which medical decisions are made, they might raise questions about who makes decisions in complex health contexts. According to Das et al [24], portals accessed by clinicians should consider whether systems are established to ensure adequate decisional support and interdisciplinary interaction. Where web portals are used in the course of clinical decision-making, it is important to ensure that the provided information is supported by appropriate expertise in the circumstance. Health portal developers may also be attentive to maintaining a balance between the aim of empowering individual patients to make decisions about their own health while protecting their capacity to access high-quality clinical support and expertise. Our findings suggest that web portals may make medical decision-making significantly more efficient for numerous stakeholders, but it is critical to ensure that such decisional resources are well calibrated to the needs and capacities of the individuals using them.

### Strengths and Limitations

This review attempts to collect preliminary data concerning proposals to implement therapeutic portals. This research encountered 3 important limitations that should be addressed in future work. First, the search terms employed in this review may not have been maximally targeted to the phenomenon under consideration. The reason for this is that the precise area of research with which we were interested remains relatively gestational and the terminology surrounding web portals that incorporate health research and patient perspectives has not yet been settled. It may also be that a number of therapeutic portals under development have not been described in published articles. This being the case, we hope that it will become increasingly commonplace for scholars and researchers to publish their experiences and perspectives on web portal design and implementation. Second, we did not specifically seek to include gray literature sources in this review. It is highly likely that some sources on therapeutic portals have been communicated outside of traditional academic settings. As our review focused on articles published in peer-reviewed periodicals, we did not assess any potentially pertinent gray literature sources. Future research on this topic should be expanded to include consideration of published works outside of traditional academic settings. Third, this review was limited by language and jurisdiction. Each of the 22 sources we reviewed was published in English and obtained from the English search databases. Moreover, most sources were written by scholars working in North America. These factors likely limited the scope of our search. Future research should explicitly engage with research published in languages other than English and by scholars in a more diverse set of jurisdictions. Despite these limitations, we believe that this study provides a useful overview of the landscape for therapeutic portals, including their specific potential objectives and legal, ethical, and policy challenges they are likely to face.

### Conclusions

This paper communicates the findings of a scoping literature review on the potential development and implementation of what we have called therapeutic portals, web systems that facilitate the sharing of health information among researchers, clinicians, patients, and the public. Such portals differ from patient portals in several important respects. First, the role of researchers directly communicating novel findings with clinicians and the public could be significantly enhanced. This approach could help promote the growth of precision medicine as a response to complex diseases. The main aim of this study was to assess the primary legal, ethical, and policy issues associated with the development and use of web portals that facilitate information sharing between researchers, clinicians, patients, and the general public. Our findings indicate that *therapeutic portals* are a potentially promising but largely underexplored area of study. Of the 22 studies reviewed, 8 (36%) contemplated therapeutic portals were defined. The portals we reviewed took a variety of forms and covered a range of medical issues. The absence of extensive literature on portals that specifically integrate uses from clinical, research, and public settings indicate that such systems have not been implemented on a large scale. We found that developing therapeutic portals would raise a number of important legal, ethical, and social issues, including those surrounding privacy and confidentiality, patient health literacy, equity, training, and decision-making. Each of these considerations should be given careful attention through the implementation of robust policy frameworks for managing the functions of novel portals, especially those that are of a form that has not yet been widely adopted. Further research is critical to better understand the perspectives of clinicians, researchers, patients, and members of the public on the contours of possible therapeutic portals. Although web portals that interface the numerous vital constituents in health research are, in principle, highly promising, any novel system must account for the various legal, ethical, and social challenges that they will present.

It is noteworthy that, although several of the papers included in the final review are several years post publication, those that more recently entered the literature express concern for the same kinds of issues and potential concerns. This indicates that the policy considerations identified here have not yet been fully addressed in policy development over the last decade. This underscores the responsibility for developers of the next generation of web portals to rededicate themselves to the challenge of implementing web tools that account for the serious legal, ethical, and social issues identified in this study.

This review is situated within a broader discussion in the literature on patients and other portals for managing health data and sharing information with patients and the public. The rapidly expanding use of web systems has generally been held out as a success that has contributed to improved outcomes and greater patient empowerment [43,44]. Our results suggest that web portals and systems are a promising mechanism for improving patient engagement, facilitating rapid research translation, and ultimately improving outcomes. There is great promise for portals that explicitly promote the sharing of basic research findings and engage with patients and the broader public. Portals of this kind would need to engage across a varied set of stakeholders with varying needs and levels of expertise. Further policy and empirical research will be necessary to develop strategies that are responsive to the unique challenges that such systems would raise. As web portals become increasingly important mechanisms for sharing health research with clinicians, patients, and the public, it is vital that these developments are met with ethical and conceptual scrutiny. The therapeutic portals presented in this paper may become a more widespread feature of precision and translational medicine. Our findings suggest that web portals are already being used to disseminate research results among clinicians, patients, and the public. However, much of the ethical and conceptual debate is framed in terms of the patient portal, a concept that does not adequately reflect the potentially broader scope of therapeutic portals. It may be useful to clarify this distinction in future research and to underscore the unique ethical, legal, and policy challenges raised when web systems are used as a platform for disseminating research to as wide an audience as possible.

## Abbreviations

AML: acute myeloid leukemia

## References

[1] Doyle D (1996). The internet and medicine: past, present, and future. Yale J Biol Med.

[2] Robinson A (2007). Revolutionizing 21st century medicine with consumer-based diagnostics and the internet. J Am Physicians Surg.

[3] Hood L, Balling R, Auffray C (2012). Revolutionizing medicine in the 21st century through systems approaches. Biotechnol J.

[4] Klein DM, Fix GM, Hogan TP, Simon SR, Nazi KM, Turvey CL (2015). Use of the blue button online tool for sharing health information: qualitative interviews with patients and providers. J Med Internet Res.

[5] Davoody N, Koch S, Krakau I, Hägglund M (2019). Accessing and sharing health information for post-discharge stroke care through a national health information exchange platform - a case study. BMC Med Inform Decis Mak.

[6] Pastorino R, De Vito C, Migliara G, Glocker K, Binenbaum I, Ricciardi W, Boccia S (2019). Benefits and challenges of big data in healthcare: an overview of the european initiatives. Eur J Public Health.

[7] Irizarry T, Dabbs AD, Curran CR (2015). Patient portals and patient engagement: a state of the science review. J Med Internet Res.

[8] Mangino D, Danis M (2020). Sharing ethics consultation notes with patients through online portals. AMA J Ethics.

[9] Daskalakis DC (2017). The electronic health record and patient portals in HIV medicine: pushing the boundaries of current ethics and stigma. Camb Q Healthc Ethics.

[10] Lau M, Campbell H, Tang T, Thompson DJ, Elliott T (2014). Impact of patient use of an online patient portal on diabetes outcomes. Can J Diabetes.

[11] Lavallée VP, Krosl J, Lemieux S, Boucher G, Gendron P, Pabst C, Boivin I, Marinier A, Guidos CJ, Meloche S, Hébert J, Sauvageau G (2016). Chemo-genomic interrogation of CEBPA mutated AML reveals recurrent CSF3R mutations and subgroup sensitivity to JAK inhibitors. Blood.

[12] Black L, Avard D, Zawati M, Knoppers B, Hébert J, Sauvageau G, Leucegene Project (2013). Funding considerations for the disclosure of genetic incidental findings in biobank research. Clin Genet.

[13] Arksey H, O'Malley L (2005). Scoping studies: towards a methodological framework. Int J Soc Res Methodol.

[14] Mays N, Roberts E, Popay J, Fulop N, Allen P, Clarke A, Black N (2001). Synthesizing research evidence. Studying the Organisation and Delivery of Health Services: Research Methods.

[15] Nordqvist C, Hanberger L, Timpka T, Nordfeldt S (2009). Health professionals' attitudes towards using a Web 2.0 portal for child and adolescent diabetes care: qualitative study. J Med Internet Res.

[16] Kaiser J (2018). A new portal for patient data. Science.

[17] Li R, Scott J, Rockhold F, Hill N, Wood J, Sim I (2018). Moving data sharing forward: the launch of the vivli platform. National Academy of Medicine.

[18] Cai L, Lin S, Girard L, Zhou Y, Yang L, Ci B, Zhou Q, Luo D, Yao B, Tang H, Allen J, Huffman K, Gazdar A, Heymach J, Wistuba I, Xiao G, Minna J, Xie Y (2019). LCE: an open web portal to explore gene expression and clinical associations in lung cancer. Oncogene.

[19] Baldwin JL, Singh H, Sittig DF, Giardina TD (2017). Patient portals and health apps: pitfalls, promises, and what one might learn from the other. Healthc (Amst).

[20] Bartonova A (2012). How can scientists bring research to use: the HENVINET experience. Environ Health.

[21] Bostrom HE, Shulaker B, Rippon J, Wood R (2017). Strategic and integrated planning for healthy, connected cities: chattanooga case study. Prev Med.

[22] Bowler L, Hong W, He D (2011). The visibility of health web portals for teens: a hyperlink analysis. Online Inform Rev.

[23] Christensen H, Murray K, Calear AL, Bennett K, Bennett A, Griffiths KM (2010). Beacon: a web portal to high-quality mental health websites for use by health professionals and the public. Med J Aust.

[24] Das A, Faxvaag A, Svanæs D (2015). The impact of an eHealth portal on health care professionals' interaction with patients: qualitative study. J Med Internet Res.

[25] Kirkpatrick BE, Riggs ER, Azzariti DR, Miller VR, Ledbetter DH, Miller DT, Rehm H, Martin CL, Faucett WA, ClinGen Resource (2015). GenomeConnect: matchmaking between patients, clinical laboratories, and researchers to improve genomic knowledge. Hum Mutat.

[26] Kohler S (2013). Can internet access growth help reduce the global burden of noncommunicable diseases?. Online J Public Health Inform.

[27] Kuijpers W, Groen WG, Loos R, Oldenburg HS, Wouters MW, Aaronson NK, van Harten WH (2015). An interactive portal to empower cancer survivors: a qualitative study on user expectations. Support Care Cancer.

[28] Maggio LA, Moorhead LL, Willinsky JM (2016). Qualitative study of physicians' varied uses of biomedical research in the USA. BMJ Open.

[29] Marrie RA, Leung S, Tyry T, Cutter GR, Fox R, Salter A (2019). Use of eHealth and mHealth technology by persons with multiple sclerosis. Mult Scler Relat Disord.

[30] McKemmish S, Manaszewicz R, Burstein F, Fisher J (2009). Consumer empowerment through metadata-based information quality reporting: the breast cancer knowledge online portal. J Am Soc Inf Sci.

[31] Melchart D, Eustachi A, Gronwald S, Wühr E, Wifling K, Bachmeier BE (2018). Introduction of a web portal for an individual health management and observational health data sciences. Patient Relat Outcome Meas.

[32] Melholt C, Joensson K, Spindler H, Hansen J, Andreasen JJ, Nielsen G, Noergaard A, Tracey A, Thorup C, Kringelholt R, Dinesen BI (2018). Cardiac patients' experiences with a telerehabilitation web portal: implications for eHealth literacy. Patient Educ Couns.

[33] Nordfeldt S, Ängarne-Lindberg T, Berterö C (2012). To use or not to use--practitioners' perceptions of an open web portal for young patients with diabetes. J Med Internet Res.

[34] Rocker C, Cappelletti L, Marshall C, Meunier CC, Brooks DW, Sherer T, Chowdhury S (2015). Use of an online portal to facilitate clinical trial recruitment: a preliminary analysis of Fox Trial Finder. J Parkinsons Dis.

[35] Sutherland JJ, Stevens JL, Johnson K, Elango N, Webster YW, Mills BJ, Robertson DH (2019). A novel open access web portal for integrating mechanistic and toxicogenomic study results. Toxicol Sci.

[36] Tomlinson C, Thimma M, Alexandrakis S, Castillo T, Dennis JL, Brooks A, Bradley T, Turnbull C, Blaveri E, Barton G, Chiba N, Maratou K, Soutter P, Aitman T, Game L (2008). MiMiR--an integrated platform for microarray data sharing, mining and analysis. BMC Bioinformatics.

[37] Moscarello T, Murray B, Reuter CM, Demo E (2019). Direct-to-consumer raw genetic data and third-party interpretation services: more burden than bargain?. Genet Med.

[38] Antonio M, Petrovskaya O, Lau F (2019). Is research on patient portals attuned to health equity? a scoping review. J Am Med Inform Assoc.

[39] Kujala S, Hörhammer I, Kaipio J, Heponiemi T (2018). Health professionals' expectations of a national patient portal for self-management. Int J Med Inform.

[40] Coughlin S, Prochaska J, Williams LB, Besenyi G, Heboyan V, Goggans S, Yoo W, De Leo G (2017). Patient web portals, disease management, and primary prevention. Risk Manag Healthc Policy.

[41] Han H, Gleason KT, Sun C, Miller HN, Kang SJ, Chow S, Anderson R, Nagy P, Bauer T (2019). Using patient portals to improve patient outcomes: systematic review. JMIR Hum Factors.

[42] Bush RA, Connelly CD, Fuller M, Pérez A (2016). Implementation of the integrated electronic patient portal in the pediatric population: a systematic review. Telemed J E Health.

[43] Kipping S, Stuckey MI, Hernandez A, Nguyen T, Riahi S (2016). A web-based patient portal for mental health care: benefits evaluation. J Med Internet Res.

[44] Irizarry T, Shoemake J, Nilsen ML, Czaja S, Beach S, Dabbs AD (2017). Patient portals as a tool for health care engagement: a mixed-method study of older adults with varying levels of health literacy and prior patient portal use. J Med Internet Res.

